# Effect of an educational intervention on nurses’ competence in activities of daily living support in end-of-life care using a pretest–posttest repeated measures design

**DOI:** 10.1186/s12904-023-01232-2

**Published:** 2023-08-22

**Authors:** Heidrun Gattinger, Stefan Ott, Carola Maurer, Brigitte Marty-Teuber, Virpi Hantikainen, André Fringer

**Affiliations:** 1 Institut of Applied Nursing Science, Eastern Switzerland University of Applied Sciences, Rosenbergstrasse 59, St. Gallen, Postfach, St. Gallen, 9001 Switzerland; 2 School of Management, Eastern Switzerland University of Applied Sciences, Rosenbergstrasse 59, St. Gallen, Postfach, St. Gallen, 9001 Switzerland; 3 Kinaesthetics Schweiz, Zürcherstrasse 46, Winterthur, 8400 Switzerland; 4 Department of Nursing Science, University of Turku, Turku, Turun yliopisto, 20014 Finland; 5 ZHAW School of Health Sciences, Institute of Nursing, Katharina-Sulzer-Platz 9, Winterthur, 8400 Switzerland

**Keywords:** Kinaesthetics, End-of-life care, Activities of daily living, Basic nursing care, Nurses’ competence

## Abstract

**Background:**

Most patients in specialized palliative care units need nursing support to perform activities of daily living (ADL), such as using a toilet or transferring out of a bed or chair. To deliver high-quality ADL support that facilitates patients’ movement and protects nurses’ musculoskeletal health, nurses need appropriate knowledge and skills. The objective of this study is to investigate the impact of education based on the “Advanced Kinaesthetics in Palliative care (AdKinPal) program” on the competence in Kinaesthetics, self-efficacy regarding ADL support in end-of-life care and musculoskeletal complaints of nurses from specialist palliative care units.

**Methods:**

A pretest–posttest repeated measures design was applied. The study took place in three specialised units for palliative care in Switzerland between June 2018 and April 2020. All the nurses who worked in participating wards (n = 62) and fulfilled the inclusion criteria were asked to participate. The intervention – the AdKinPal program – is an education-based training program conducted for six months. We took measurements using self-administered questionnaires at three points before and after the intervention. Using descriptive statistics, repeated measurement analysis of variance (ANOVA) and independent-samples t-tests, we analysed the participants’ demographic characteristics as well as developments over time and relationships between the three outcome variables: Kinaesthetics competence, self-efficacy regarding ADL support in end-of-life care and musculoskeletal complaints.

**Results:**

Fifty-nine nurses and one physiotherapist participated, and 38 participants (63%) responded to all three questionnaires. The AdKinPal training improved the nurses’ perceived Kinaesthetics competence and self-efficacy regarding ADL support in end-of-life care. Participants who reported lower back, neck or shoulder pain had a significantly lower Kinaesthetics competence.

**Conclusions:**

The AdKinPal program can raise nurses’ Kinaesthetics competence. Thereby, patients’ autonomy and quality of life could be supported, and symptom management could be enhanced in a holistic manner. Furthermore, the AdKinPal program fosters nurses’ self-efficacy in ADL support in end-of-life care. A strong sense of self-efficacy enhances professional well-being in many ways. Additionally, the nursing staff’s musculoskeletal health can be promoted by enhancing their Kinaesthetics competence.

**Trial registration:**

DRKS00015908. Registration Date 23.11.2018.

## Background

In high-income countries, a majority of people (73%) die in an institution, such as a hospital or nursing home [1]. Health care professionals in specialised palliative care units are frequently confronted with caring for people at the end of their life. End-of-life care is defined in this study based on the narrow definition of end-of-life [2] as a period where the person is unable to care for themselves and the disease may be rapidly progressing (Karnofsky Performance Status Index ≤ 50) [3].

Patients at the end of life with specialised palliative care needs experience a heavy symptom burden. A systematic review identified symptom prevalence among adults with advanced cancer and non-cancer disease, such as end-stage renal disease, heart disease, chronic obstructive pulmonary disease or Parkinson’s disease. Symptom prevalence ranges from 13 to 100% for fatigue, 11 to 98% for pain, 11 to 98% for breathlessness, 2 to 78% for nausea, 2 to 70% for confusion and 4 to 65% for constipation [4]. In addition, a significant number of patients have physical limitations because of illness progression, chemotherapy, radiotherapy, surgery, and other complex treatments. For persons diagnosed with a terminal illness, autonomy and maintenance of independence are highly important [5, 6].

Most of these patients require nursing support to perform basic self-care activities – known as activities of daily living (ADL) – such as bathing, dressing, using a toilet or transferring out of a bed or chair. Self-care activities can be affected by symptoms such as pain, fatigue or breathlessness. The kind of support provided by nurses while supporting ADL can help relieve pain [7, 8]. To manage symptoms such as fatigue and dyspnoea, energy conservation and simplification techniques are central while supporting patients with their daily activities. This includes pacing activities, taking frequent rests and avoiding strenuous motions. A balance must be found between increasing rest to alleviate asthenia and supporting independence and autonomy [7, 9]. Additionally, appropriate movement and positioning support can convey orientation and safety and prevent pressure ulcers [7, 10].

To deliver high-quality ADL support, nurses need appropriate knowledge and skills [11]. However, nurses have uncertainties regarding how to provide high-quality ADL support for persons with specialised palliative care needs, especially regarding moving or positioning the person such that they do not experience much pain and dyspnoea [8, 12].

Nurses offering specialised palliative care are particularly at risk of experiencing stress [13, 14]. However, strengthening nurses’ competence and confidence improves their self-efficacy [15]. Subsequently, higher self-efficacy buffers the impact of perceived stressful encounters on their professional quality of life [16]. Finally, nurses frequently suffer from work-related pain and musculoskeletal disorders while providing physical care, such as moving a patient in and out of bed. Prevalence figures reported pain in the last 3–6 months as follows: 44% had lower back pain, 44% had shoulder pain, 48% had neck pain, 21% had upper extremity pain and 38% had lower extremity pain [17]. Therefore, nurses need to gain competence in moving and transferring patients to prevent endangering their own health [18].

The need for nurses’ competence development to improve their ADL support skills has been acknowledged [19, 20]. Different training approaches exist, such as Natural Mobility [21], PERSAMO (PERson-centred and SAfe MObility care) [22] or the Bobath concept for neurologically impaired patients [23]. The first two approaches have not yet been adequately researched, while the Bobath concept is a disease-specific concept. However, Kinaesthetics is a training concept that enhances nurses’ competence in daily activity support and is effective in improving patients’ functionality and quality of life [11, 24] as well as nurses’ musculoskeletal health [11]. Kinaesthetics training aims to increase the quality of interaction and movement of the patient and the nurse in everyday activities. Nurses are enabled to adapt the direct interactions via touch and movement in everyday support to the situation mindfully and individually. Thereby, patients’ self-determination, independence and self-care are supported, and nurses’ musculoskeletal health is enhanced [11, 25]. In German-speaking countries (Germany, Austria, and Switzerland), Kinaesthetics training is integrated in vocational nursing education. However, in practice, nurses’ competence in ADL support based on Kinaesthetics varies significantly, and competence development is not supported systematically [26, 27]. A systematic approach is needed to gain favourable patient and nursing outcomes [24, 28, 29].

### Aim and research questions

To address the demands of competence development in end-of-life care mentioned above, our research team developed an education-based intervention for nurses working in specialised palliative care settings, the Advanced Kinaesthetics in Palliative care (AdKinPal) program (see Intervention). The development of this intervention was based on the curricula of Kinaesthetics [30, 31], previous studies [11, 12] and a workshop and interviews with 14 experts (nurses with Kinaesthetics trainer certificate and palliative care experience).

The objective of the study is to explore and investigate the impact of education based on the AdKinPal program on the competence in Kinaesthetics, self-efficacy regarding ADL support in end-of-life care and musculoskeletal complaints of nurses from specialist palliative care units. In addition, patient data on symptom prevalence was collected (publication in progress) and a process evaluation was conducted [32]. In this article, we focus on the following research questions:

- Does the AdKinPal program improve nurses’ competence in Kinaesthetics and self-efficacy regarding ADL support in end-of-life care?
- Does the AdKinPal program reduce nurses’ musculoskeletal complaints?

## Methods

**Fig. 1:**
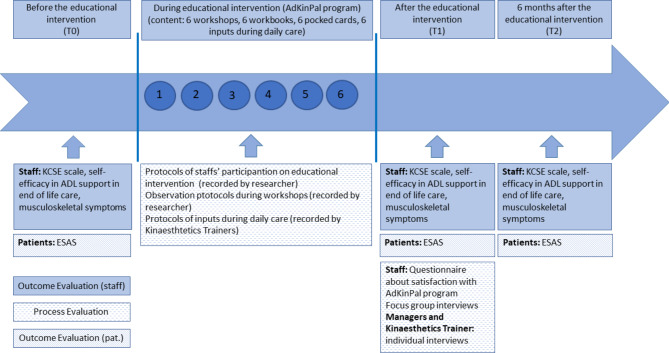
Timeline of the data collection in the AdKinPal study; process and outcome evaluation of patients’ data are not part of this article KCSE: Kinaesthetics Competence Self-Evaluation, ESAS: Edmonton Symptom Assessment System.

A phase II exploratory study [33] using a pretest–posttest repeated-measures design, including a process evaluation [34], was applied. The measurement time points are before (T0) and after the education-based intervention (T1) and six months after the intervention was finished (T2) (Fig. 1).

### Setting and participants

The study took place in three specialised units for palliative care in three community hospitals in the German-speaking part of Switzerland. All the nurses who worked in participating wards and fulfilled the inclusion criteria were asked to participate. The inclusion criteria were as follows:

- Registered nurses (diploma or bachelor’s degree), licenced practical nurses, nursing aids working in direct care.
- Aged 18 years or above.
- Informed consent.

Furthermore, the participation in the study was open to physiotherapists who regularly worked in the palliative care units, and who also had the task of helping patients with support in activities of daily living.

The sample size was calculated for repeated measurement comparison of the primary outcome (competence in Kinaesthetics) [26]. With an assumed effect size of 1 (on the 4–16 points Kinaesthetics Competence Self-Evaluation [KCSE] scale) and a conservatively estimated standard deviation of 2.5, a total sample size of 49 nurses was required (level of significance α = 0.05 and power 80%). To account for dropouts and missing values (20%) or repeated measurement data, the starting sample required around 60 nurses.

### Intervention

The intervention was an education-based training program conducted for six months. Some of the key features of the multi-faceted program were workshops, workbooks and small note cards and inputs during daily care (Table 1). Every month, a “theme of the month”, for example, mindfulness movement, was introduced during a workshop. A workbook was handed out to all the nurses. Participants were also given small note cards, which could be carried in their work clothes, to remember and integrate the theme of the month in their daily work. Furthermore, nurses were offered individual coaching during daily care situations to support the implementation of the training content.

Nurses with a level 3 Kinaesthetics trainer certificate conducted the workshops and individual coaching. Level 3 Kinaesthetics trainers have a minimum of 1366 h of training and four years of experience with Kinaesthetics [35]. The intervention facilitators also had several years of experience in palliative care.

**Table 1:** AdKinPal program for palliative care nurses

Component of the intervention	Thematic workshops (theme of the month)	Workbooks and small note cards	Inputs during daily care
Content/ Procedure	∎ Mindfulness movement ∎ Quality of the interaction of touch and movement ∎ Body awareness: Experience of characteristics and functions of body structure ∎ Recognition of movement possibilities ∎ Positioning support and arrangement of the environment ∎ Movement till the last breath	Workbooks and small note cards that could be tucked into the nurses’ work clothes for individual study and reflective practice. For example, they included reflective questions about the theme of the month.	Individual coaching for nurses: Kinaesthetics trainers accompanied the nurses during daily care situations and supported reflective practice.
Frequency	1 per month over 6 months	1 per month over 6 months	1 per month over 6 months
Length	2 h	varied per person	4–6 h

### Recruitment and data collection

The investigator informed the nurses about the study and informed consent was obtained. Data were collected from the participating nurses with paper and pencil questionnaires distributed by a member of the research team. The completed questionnaires were returned to the head nurse in an enclosed envelope and then sent to the research team.

### Variables and measurement

The data were collected using three instruments: the KCSE scale (28 items) [36], nurses’ self-efficacy in ADL support in end-of-life care scale (14 items) and the Nordic questionnaire for analysis of musculoskeletal symptoms (9 items) [37]. The nurses’ sociodemographic data were collected with ten items concerning age, gender, employment status, nursing education, experience in palliative care and training in palliative care and Kinaesthetics. The KCSE scale is based on a concept development study aiming to describe nurses’ competence in Kinaesthetics [11] and contains four subscales: knowledge of Kinaesthetics, self-perceived skills of Kinaesthetics, attitude and dynamic state (Table 2).

**Table 2:** Content of the KCSE scale [36]

Subscale	Knowledge	Skills	Attitude	Dynamic state
Content	Knowledge of the theoretical principles of Kinaesthetics, such as how the movement elements of time, space and effort are related to each other.	Skills regarding interaction, movement support of the person in need of care, nurses’ own movement, and the adaptation of the environment in such a way that the movement of the person in need of care is promoted.	An attitude that recognises the learning and development process of each person and uses everyday support situations as learning opportunities.	Dynamic state refers to the nurses’ ability to reflect and their motivation to further develop their Kinaesthetic competence in everyday care.

The items have four response options for agreement (disagree, somewhat agree, agree, strongly agree), frequency (never, sometimes, almost every time, every time) and quality (not at all, somewhat, good, very good). The KCSE scale has a good content validity index (0.93) and internal consistency (Cronbach’s alpha = 0.91) [36].

The nurses’ self-efficacy in ADL support in end-of-life care scale was developed by the research team based on Banduras’ guide for constructing self-efficacy scales [38]. The scale contains 14 items, where nurses rate their self-efficacy in ADL support in end-of-life care on a scale from 0 (not capable) to 10 (exceptionally capable). Here are two examples of the items: “I am able to support a patient with pain to find a pain-reducing position” and “I am able to support a patient with breathlessness during daily care activities”. The scale was tested on face and content validity by six experts.

The Nordic questionnaire for the analysis of musculoskeletal symptoms covers nine symptom sites (neck, shoulders, elbows, wrist/hands, upper back, lower back, hips/thighs, knees and ankles/feet). The respondents were asked if they experienced any musculoskeletal trouble that prevented normal activity (the answer categories were yes and no). The Nordic questionnaire for analysis of musculoskeletal symptoms has good test–retest reliability, and its validity among different occupational groups [37] and nurses [39] has been established. We used the German version [40] with a recall period of 3 months. Data collection took place between June 2018 and April 2020.

### Data management and analysis

The study data were recorded with paper Case Report Forms (CRF). CRF did not contain any person identifying information, but appropriate coded identification was used. Data were checked for coding errors and entered into IBM SPSS Statistics, version 25. For 10% of the data, a double data entry was performed to check the accuracy of the data entry [41]. The verification of the double-entered data records showed only slight deviations (less than 0.5%), indicating high data integrity. The CRF and electronic data were stored according to the law [42].

The data were analysed as follows: In the KCSE scale, single items scored between 1 and 4, with higher scores indicating higher self-evaluated competence in Kinaesthetics. For the KCSE subscales of knowledge, skills, attitude and dynamic state, mean scores were calculated (range 1–4). The total score was calculated by adding the mean scores of the subscales (range 4–16) [36].

For the nurses’ self-efficacy in ADL support in palliative care, assessed by the ADL support in Palliative Care Self-Efficacy Scale (ADL-PC-SES), the mean score of all the items was calculated, which ranges from 0 to 10.

Data regarding musculoskeletal complaints, assessed by the Nordic questionnaire for the analysis of musculoskeletal symptoms, were analysed separately for each body region. The total score of all the complaints (sum of the complaints) for each participant at each time point was calculated.

The sociodemographic characteristics of the study participants were evaluated by descriptive statistics according to the underlying data scales (frequencies for qualitative data, range, mean values and standard deviations for metric data).

The nurses’ competence in Kinaesthetics, measured by the total score and subscores, and the nursing staff’s self-efficacy were characterized by descriptive statistics (mean and standard deviation) and analysed using a repeated measurement ANOVA, accounting for the longitudinal nature of the data. Similarly, the nurses’ musculoskeletal complaints were characterized by frequency and analysed using a repeated measurement ANOVA. To better handle missing values, we used the SPSS procedure MIXED. The assumption of sphericity was tested using Mauchly’s test and, in case of violation, the correction by Greenhouse-Geisser was taken into consideration. The relationship between the nurses’ competence in Kinaesthetics (total score) and nursing staff’s self-efficacy was tested using linear regression. Furthermore, the nursing staff was grouped by the prevalence of certain symptoms (neck pain, shoulder pain, lower back pain) and tested for differences in their Kinaesthetics competence (total score and subscores) using independent-samples t-tests. Additionally, relevant assumptions were tested for their fulfilment. All the tests were performed at a level of significance of 0.05. The statistical analysis was provided by a statistician using IBM SPSS Statistics, version 25.

### Ethical considerations

The study was carried out following the protocol and principles enunciated in the current version of the Declaration of Helsinki, the guidelines of Good Clinical Practice, and Swiss Law and the Swiss regulatory authority’s requirements. The ethics commission Ostschweiz has approved the study protocol (EKOS 2017 − 00721).

## Results

**Fig. 2:**
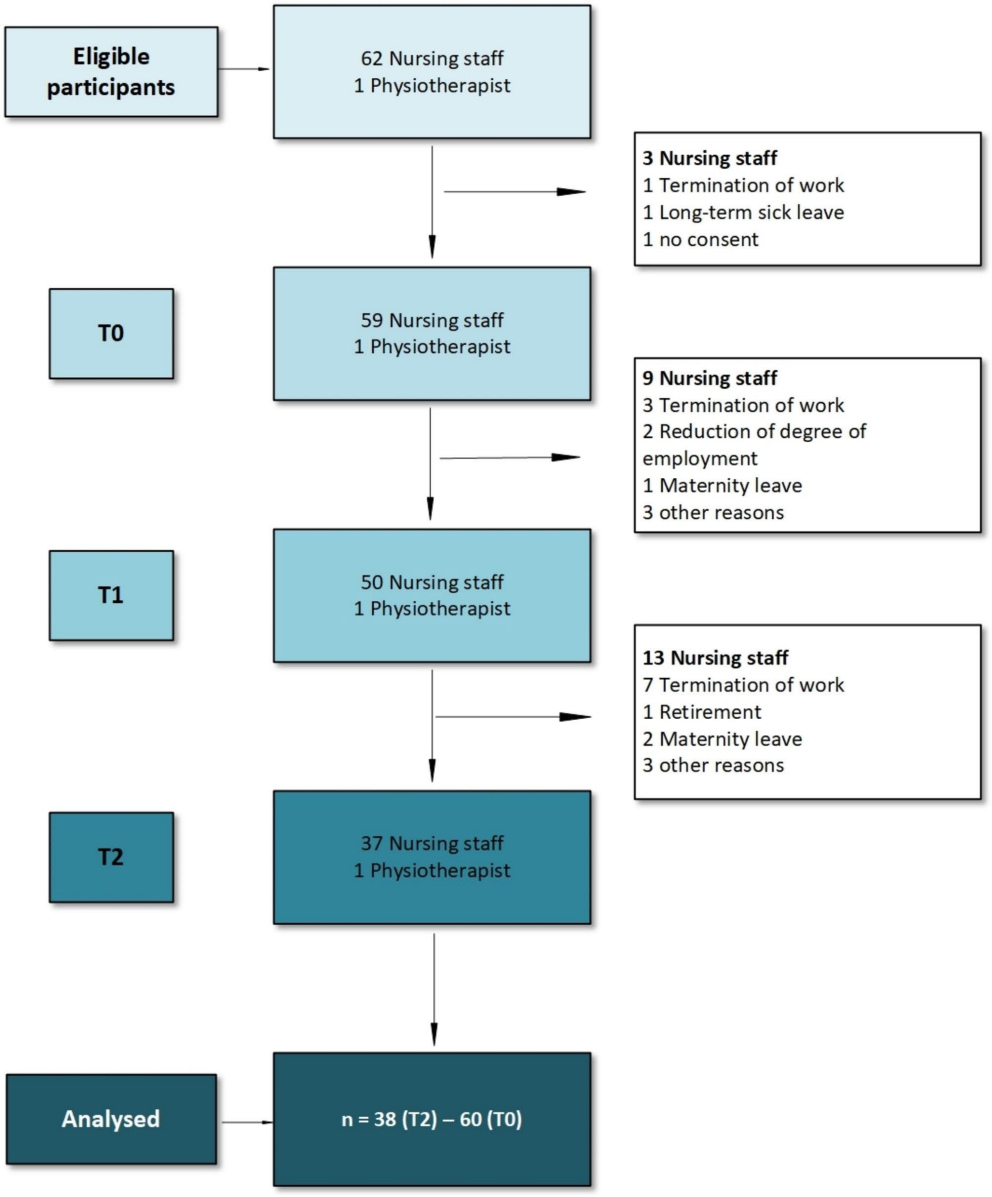
Flow diagram of the study participants

Of the eligible nursing staff (n = 62), 59 nurses (95%) and one physiotherapist signed the informed consent, and 38 participants (63%) responded to all three questionnaires (Fig. 2). The participants’ mean age was 40.83 years. Their mean experience in palliative care was 6.79 years. Most of the participants were registered nurses (74%), followed by licensed practical nurses (12%) and nurse assistants (12%). They have worked for an average of 5.12 years in the current institution. 32% had passed an advanced Kinaesthetics training course and 43% a basic one, and 20% had received no standard Kinaesthetics training (Table 3).

**Table 3:** The sociodemographic, employment and competence characteristics of the study participants (n = 60)

Characteristics	n	%	*M* (*SD)*		Range
**Age** (years)	60		40.83 (12.12)		21–67
**Gender** Female Male	56 4	93% 7%			
**Educational level** Registered nurse (Diploma, BScN) Licenced practical nurse Nurse assistant Physiotherapist (MSc)	45 7 7 1	74% 12% 12% 2%			
**Year of graduation** 1970–1990 1991–2005 2006–2010 Since 2011 Missing values	14 15 12 18 1	23% 25% 20% 30% 2%			
**Length of experience in palliative care** (years)	60		6.79 (5.00)		0.17–20
**Length of experience in the current unit** (years) Missing values	58 2		5.12 (3.62)		0.17–18
**Additional training in palliative care** Yes No	39 21	65% 35%			
**Employment status (full time equivalent)** 0.9–1 FTE 0.7–0.8 FTE 0.5–0.6 FTE 0.3–0.4 FTE Missing values	22 19 12 6 1	37% 31% 20% 10% 2%			
**Standard Kinesthetics training** No training Basic training course Advanced training course Peer tutoring training Trainer education I	12 26 19 2 1	20% 43% 32% 3% 2%			

M: mean value, SD: standard deviation, FTE: full time equivalent

### Nurses’ Kinaesthetics competence

The mean total score of the nurses’ Kinaesthetic competence was 11.27 at T0, 11.99 at T1 and 11.86 at T2. The nursing staff’s level of Kinaesthetic competence (KCSE total score) significantly increased over time, with F = 14.70 (2, 50) p = 0.000. Except for the subscore attitude, a significant increase was observed in the subscores of dynamic state (F = 6.42 (2, 46) p = 0.003), knowledge (F = 19.19 (2, 51) p = 0.000) and skills (F = 10.37 (2, 47), p = 0.000) between the preintervention and postintervention phase (Table 4).

**Table 4:** Development of nursing staff’s Kinaesthetics competence before intervention (T0), after intervention (T1) and at follow-up (T2) (n = 60)

KCSE scale	T0		T1		T2		ANOVA results		
	M	SD	M	SD	M	SD	F	df	p
Total score	11.27	1.44	11.99	1.42	11.86	1.72	14.70	2, 50	0.000
Attitude	3.42	0.35	3.38	0.42	3.38	0.51	0.16	2, 52	0.855
Dynamic state	2.85	0.40	2.30	0.35	2.91	0.45	6.42	2, 46	0.003
Knowledge	2.39	0.59	2.77	0.55	2.78	0.56	19.19	2, 51	0.000
Skills	2.61	0.51	2.84	0.45	2.79	0.52	10.37	2, 47	0.000

M: mean value, SD: standard deviation, F: F-statistic, df: degrees of freedom, p: p-value

### Nurses’ self-efficacy in ADL support in palliative care

The mean total score of nurses’ self-efficacy in ADL support was 7.22 at T0, 7.61 at T1 and 7.67 at T2. The nursing staff’s level of self-efficacy in ADL support in palliative care (ADL-PC-SES total score) significantly increased over time, with F = 5.46 (2, 51) p = 0.007 (Table 5).

**Table 5:** Development of the nursing staff’s self-efficacy in ADL support before intervention (T0), after intervention (T1) and at follow-up (T2) (n = 59)

ADL-PC-SES	T0		T1		T2		ANOVA results		
	M	SD	M	SD	M	SD	F	df	p
Total score	7.22	1.18	7.61	1.05	7.67	1.19	5.46	2, 51	0.007

M: mean value, SD: standard deviation, F: F-statistic, df: degrees of freedom, p: p-value

Linear regressions were calculated to investigate the interrelationship between the nursing staff’s self-efficacy in ADL support and their Kinaesthetic Competence (KCSE total score) at the three time points (T0, T1, T2). Significant relationships could be found for all three points in time: T0 (F (1, 57) = 13.47, β = 0.36, p = 0.001, 95% CI [0.16, 0.55]), with an R^2^ of 0.19; T1 (F (1, 49) = 13.72, β = 0.36, p = 0.001, 95% CI [0.16, 0.55]), with an R^2^ of 0.2; T2 (F (1, 39) = 6.99, β = 0.27, p = 0.012, 95% CI [0.06, 0.48]), with an R^2^ of 0.15.

### Nurses’ musculoskeletal complaints

**Fig. 3:**
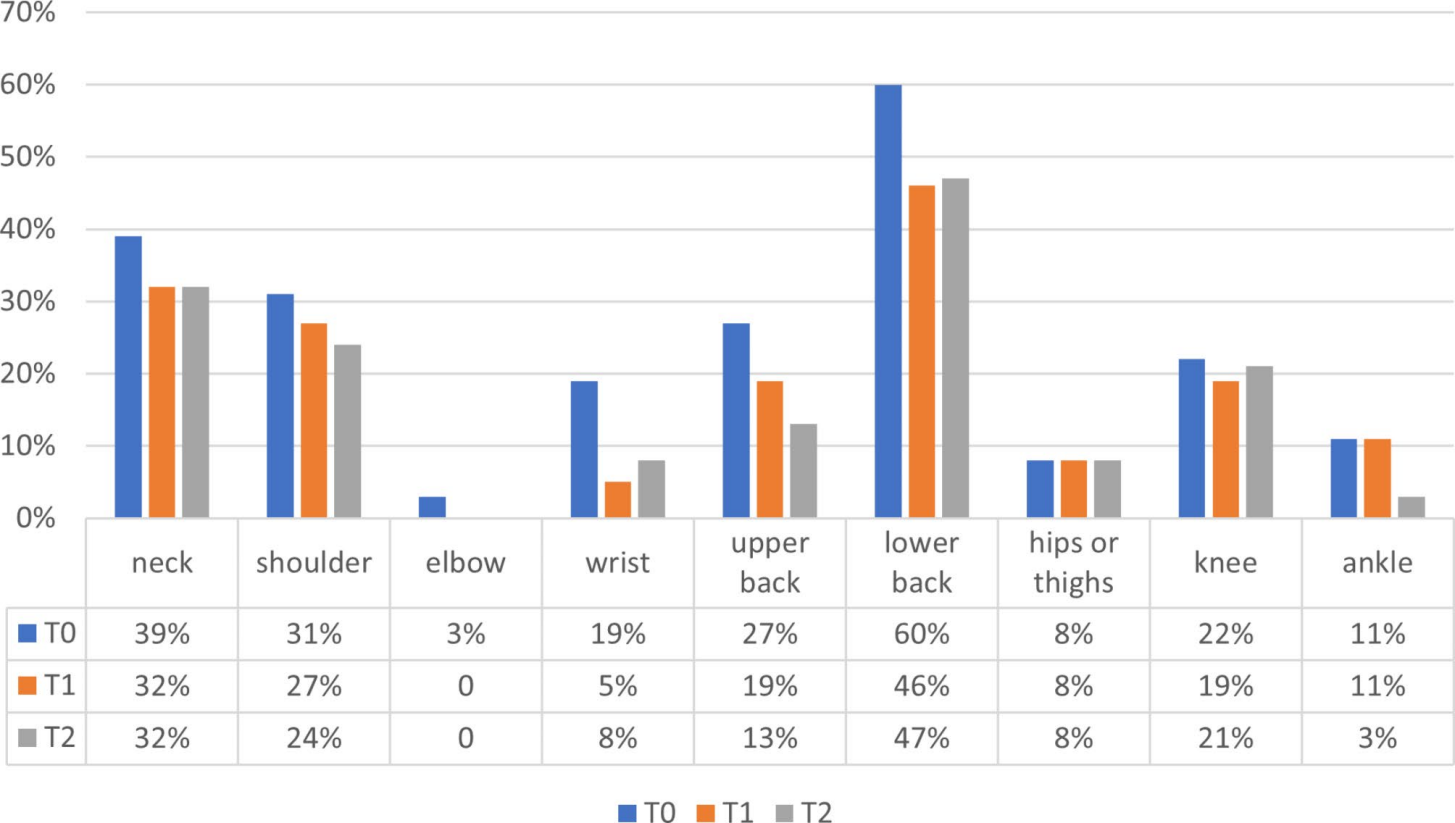
Incidence of the nursing staff’s musculoskeletal complaints before intervention (T0), after intervention (T1) and at follow-up (T2) (on the data available during all three time points, n = 36–38)

The mean total score of musculoskeletal complaints was 2.22 at T0, 1.76 at T1 and 1.54 at T2. The incidence of nursing staff’s musculoskeletal complaints over the three measurement time points is displayed in Fig. 3. No significant reduction was observed in the musculoskeletal complaints over time and the sum of musculoskeletal complaints.

Even though we couldn’t find a significant reduction in musculoskeletal complaints over time, an obvious relationship exists between nurses’ Kinaesthetics competences and musculoskeletal complaints for body parts where pain was most frequently reported (lower back, neck and shoulder). Participants who reported pain had a significantly lower KCSE total score (lower Kinaesthetics competence): pain in the lower back (t (148) = 2.49, p = 0.014), neck (t (147) = 2.98, p = 0.003) and shoulder (t (145) = 2.04, p = 0.043). See Table 6 for the complete results, including the subscores. For all three body parts, the subscore “skills” is significantly higher in the group that did not report pain, whereas we did not find any differences in the subscore “attitude”.

**Table 6:** T-test results comparing Kinaesthetics competence (KCSE total and subscores) and pain in the lower back, neck and shoulder

KCSE scale	No lower back pain (n = 73)		Lower back pain (n = 77)			
	M	SD	M	SD	t (df)	p
Total score	12.0	1.63	11.38	1.41	2.49 (148)	0.014
Attitude	3.42	0.40	3.37	0.44	0.80 (148)	0.425
Dynamic state	2.99	0.41	2.85	0.39	2.20 (148)	0.029
Knowledge	2.75	0.65	2.52	0.52	2.40 (148)	0.018
Skills	2.84	0.53	2.65	0.46	2.35 (148)	0.020
	**No neck pain (n = 92)**		**Neck pain (n = 57)**			
	M	SD	M	SD	t (df)	p
Total score	11.96	1.59	11.20	1.39	2.98 (147)	0.003
Attitude	3.41	0.41	3.38	0.43	0.45 (147)	0.653
Dynamic state	3.0	0.40	2.79	0.37	3.17 (147)	0.002
Knowledge	2.74	0.62	2.44	0.54	3.02 (147)	0.003
Skills	2.82	0.54	2.60	0.42	2.81 (147)	0.006
	**No shoulder pain (n = 105)**		**Shoulder pain (n = 42)**			
	M	SD	M	SD	t (df)	p
Total score	11.85	1.58	11.27	1.44	2.04 (145)	0.043
Attitude	3.43	0.38	3.31	0.51	1.53 (145)	0.129
Dynamic state	2.97	0.41	2.79	0.35	2.48 (145)	0.014
Knowledge	2.66	0.62	2.54	0.56	1.06 (145)	0.291
Skills	2.79	0.53	2.63	0.44	1.76 (145)	0.080

M: mean value, SD: standard deviation, t: t-statistic, df: degrees of freedom, p: p-value

## Discussion

This study was the first to evaluate the outcome of the AdKinPal program on palliative care nurses’ competence in Kinaesthetics, self-efficacy regarding ADL support in end-of-life care and musculoskeletal complaints. The results demonstrate that the AdKinPal training improves both nurses’ perceived Kinaesthetics competence and self-efficacy regarding ADL support in end-of-life care.

A higher Kinaesthetic competence score means that the participants better understand human movement support, have further developed their skills to be attentive during interaction via touch and movement and adapt the support to the individual situation. They better understand how to support a care-dependent person in a way that facilitates their own movement, and they adjust the physical environment to enhance the independent movement of the care-dependent person. In addition, they better understand the links between movement support and symptom management: for example, how to support movement or position to reduce pain or breathlessness. These are central elements for providing good “physical care” or “fundamental care”, as it is called in other studies [43, 44].

To our knowledge, no other studies exist on the development of Kinaesthetics competence in palliative or end-of-life care. Findings from a study on the long-term care setting show higher Kinaesthetic competence levels in the sample of nursing home staff [26]. However, this sample also had a higher level of standard Kinaesthetics training (9% no training, 38% basic training, 37% advanced training, 13% peer tutoring training and 3% trainer education) compared to this study’s sample (20% no training, 43% basic training, 32% advanced training, 3% peer tutoring training and 2% trainer education). Higher Kinaesthetics competence is associated with having passed standard Kinaesthetics training courses and having completed additional Kinaesthetics training in the last twelve months [26]. Nevertheless, the AdKinPal programme was able to significantly increase the Kinaesthetics competence of the palliative care nurses. Additionally, the process evaluation showed that the nurses were highly satisfied with the programme, they gained a variety of insights and they changed their behaviour; for example, they could now support a patient to sit up at an appropriate speed so that the person could actively participate [32].

Furthermore, our findings show that higher Kinaesthetic competence is correlated with higher self-efficacy regarding ADL support.

The participants’ self-efficacy regarding ADL support in end-of-life care was high before the intervention started, which is not surprising since the participants had an average of 6.8 years of experience in palliative care and 65% had completed further training in this field. Nevertheless, their self-efficacy increased significantly over time. The results show that nurses felt more confident in supporting patients with respiratory distress or pain in ADL or in advising and guiding relatives on ADL support. This is an important aspect, especially when patients are discharged and relatives need guidance on how to provide good basic care [45].

An important result is the correlation between higher Kinaesthetic competence and fewer musculoskeletal complaints. It indicates how to address the pressing problem of musculoskeletal complaints among nurses. Also, our study had a high number of nurses with musculoskeletal complaints, especially in the lower back, neck and shoulders. The nursing profession is one of the riskiest occupations for lower back pain. The primary cause for work-related musculoskeletal disorders in nursing is patient-handling tasks such as lifting, transferring, and repositioning of patients [46]. Thus, the AdKinPal Program also focused on enhancing nurses’ competence in increasing their own body awareness, that is, in recognising their own body’s tensions when they start to lift a patient and react accordingly, such as by rolling the patient instead of lifting them or adapting one’s own movement.

Although the AdKinPal program has been developed for nurses, it may be suitable for other healthcare professionals, specifically for physiotherapists and occupational therapists, who support palliative care patients with activities of daily living.

### Strengths and limitations of this research

The AdKinPal program addresses both individual and organisational aspects, affecting all nurses and, consequently, patients within the uptake area. Therefore, a control in the same unit is not feasible [47]. By applying this study design, we aimed to get more knowledge about the effects and feasibility of this intervention for a subsequent cluster randomised trial. The process evaluation also gave us indications on how to improve the program [32]. In the course of the study, we had a dropout of 37% (22 from 60 study participants). During the follow-up period, one participating ward experienced organisational changes due to the onset of the corona pandemic, which led to nurses leaving the ward. This probably had no impact on the study’s results since the dropouts had characteristics similar to those who remain in the study. However, a high nursing turnover could influence the sustainability of the intervention [32]. To evaluate their Kinaesthetics competence, we used a self-assessment approach. The validity/reliability of self-assessed measures is debated, although it has been reported as the most common form of competence assessment [48]. However, in future studies, we recommend also using an external assessment of nurses’ competence in Kinaesthetics to reinforce or dispel the nursing staff’s perceptions [49].

### Implications for practice and research

The complexity of care at the end-of-life is well recognized. The integration of non-pharmacological and pharmacological management is required [50]. Patients and their families consider good physical care, symptom management and integrated care to be the most important elements of end-of-life care within the hospital setting. Further, the quality of physical care is reflected in how independence is supported, such as when the care-dependent person is enabled to be in a position that allows independent eating [51]. To provide good physical care and symptom management, nurses’ competence in supporting patients in daily activities is crucial. In contrast, incompetent ADL support may result in severe negative consequences to both care-dependent persons [18] and nurses through pain and bruises and back injuries and musculoskeletal strain, respectively [52]. Therefore, it is important to ensure competence development of the nursing staff in ADL support. This study contributes new knowledge on the subject of good physical care for patients with end-of-life care demands.

More research should be conducted to explore competence development, its influencing factors and the connection between the nursing staff’s Kinaesthetics competence and patients’ outcomes, such as autonomy and self-efficacy in daily activities, and subsequently, their quality of life.

## Conclusions

The AdKinPal program could increase nurses’ Kinaesthetics competence. Thereby, patients’ autonomy and quality of life could be supported, and symptom management could be enhanced in a holistic manner. Furthermore, the AdKinPal program fosters nurses’ self-efficacy in ADL support in end-of-life care. A strong sense of self-efficacy enhances professional well-being in many ways. Additionally, nurses’ musculoskeletal health can be promoted by enhancing the nursing staffs’ Kinaesthetics competence.

The AdKinPal programme has the potential to be transferred to other areas where end-of-life care is delivered, such as in nursing homes or home care.

## Data Availability

The study data are securely stored under lock and key at Estern Switzerland University of Applied Sciences, St. Gallen, Switzerland. We do not have permission from the Ethical Committee to release or share the data; thus, we cannot make it available in the public domain. However, the datasets used and analyzed during the current study are available from the corresponding author upon reasonable request.

## List of abbreviations

ADL: Activities of Daily Living
ADL-PC-SES: Activities of Daily Living Palliative Care Self-Efficacy Scale
ANOVA: Analysis of variance
CRF: Case Report Forms
ESAS: Edmonton Symptom Assessment System
KCSE: Kinaesthetics Competence Self-Evaluation
